# Hypoxia Induced Aggressiveness of Prostate Cancer Cells Is Linked with Deregulated Expression of VEGF, IL-6 and miRNAs That Are Attenuated by CDF

**DOI:** 10.1371/journal.pone.0043726

**Published:** 2012-08-27

**Authors:** Bin Bao, Aamir Ahmad, Dejuan Kong, Shadan Ali, Asfar S. Azmi, Yiwei Li, Sanjeev Banerjee, Subhash Padhye, Fazlul H. Sarkar

**Affiliations:** 1 Department of Pathology, Karmanos Cancer Institute, Wayne State University, Detroit, Michigan, United States of America; 2 Department of Oncology, Karmanos Cancer Institute, Wayne State University, Detroit, Michigan, United States of America; 3 ISTRA, Department of Chemistry, MCE Society’s Abeda Inamdar Senior College of Arts, Science and Commerce, Pune, India; University of Nebraska Medical Center, United States of America

## Abstract

Tumor hypoxia with deregulated expression of hypoxia inducing factor (HIF) and its biological consequence leads to poor prognosis of patients diagnosed with solid tumors, resulting in higher mortality, suggesting that understanding of the molecular relationship of hypoxia with other cellular features of tumor aggressiveness would be invaluable for developing newer targeted therapy for solid tumors. Emerging evidence also suggest that hypoxia and HIF signaling pathways contributes to the acquisition of epithelial-to-mesenchymal transition (EMT), maintenance of cancer stem cell (CSC) functions, and also maintains the vicious cycle of inflammation, all of which contribute to radiation therapy and chemotherapy resistance. However, the detailed mechanisms by which hypoxia/HIF drive these events are not fully understood. Here, we have shown that hypoxia leads to increased expression of VEGF, IL-6, and CSC marker genes such as Nanog, Oct4 and EZH2, and also increased the expression of miR-21, an oncogenic miRNA, in prostate cancer (PCa) cells (PC-3 and LNCaP). The treatment of PCa cells with CDF, a novel Curcumin-derived synthetic analogue previously showed anti-tumor activity *in vivo*, inhibited the productions of VEGF and IL-6, and down-regulated the expression of Nanog, Oct4, EZH2 mRNAs, as well as miR-21 under hypoxic condition. Moreover, CDF treatment of PCa cells led to decreased cell migration under hypoxic condition. Taken together, these results suggest that the anti-tumor effect of CDF is in part mediated through deregulation of tumor hypoxic pathways, and thus CDF could become useful for cancer therapy.

## Introduction

Prostate Cancer (PCa) is the most commonly diagnosed cancer in men and it is the second leading cause of cancer death in the USA [1]. Most PCa patients are treatable, but the patients usually die due to drug resistance and metastatic disease. Thus, there is a dire need for the development of novel strategies by which drug resistance and metastatic disease could be controlled with novel agents that may improve the treatment outcome. Hypoxia is one of the fundamental biological phenomena that are intricately associated with the development and aggressiveness of a variety of solid tumors including PCa. Hypoxia-inducible factors (HIF) function as a master transcription factor, which regulates hypoxia responsive genes and have been recognized to play critical roles in tumor invasion, chemo-radiation resistance, and increased cell proliferation, survival, angiogenesis and metastasis [2]; [3]. Therefore, tumor hypoxia with deregulated expression of HIF and its biological consequence leads to poor prognosis of patients diagnosed with solid tumors, resulting in higher mortality, suggesting that understanding of the molecular relationship of hypoxia with other cellular features of tumor aggressiveness would be invaluable for developing newer targeted therapy for solid tumors.

It has been well recognized that cancer stem cells (CSCs) and epithelial-to-mesenchymal transition (EMT) phenotypic cells are associated with therapeutic resistance and contributes to aggressive tumor growth, invasion and metastasis, and are believed to be the cause of tumor recurrence [4]. Emerging evidence suggest that hypoxia and HIF pathway enhance the phenotypes and functions of CSCs and EMT [5]–[9], contributing to tumor aggressiveness, which could also be due to deregulation of microRNAs (miRNAs). The miRNAs are known to play critical roles in a wide array of biological processes, including cell differentiation, proliferation, cell death, metabolism and energy homeostasis [10]; [11]. Accumulating evidence has suggested that miRNAs might have an important role in the development and progression of tumors. The altered expression of miRNAs has been associated with clinical prognosis of tumor, resistance to chemo-radiation therapy, and tumor recurrence [12]–[14]. A large number of miRNAs have been reported to be responsive to hypoxia and HIF pathway in a wide range of cells and tissues including cancer cells [15]–[18]. It has been reported that hypoxia causes decreased expression of miR-101, a potential anti-oncogenic miRNA, and increased expression of miR-21 and miR-210, oncogenic miRNAs in various cancers including PCa [13]; [19]. Thus, hypoxia-mediated deregulation of miRNAs may play important roles in tumor aggressiveness mediated through the regulation of cellular signaling pathways including HIF pathway. Therefore, targeting these hypoxia-mediated miRNAs using novel agents may provide innovative therapeutic strategy for the prevention and/or treatment of PCa.

Here, we have examined the effects of hypoxia on cell migration, invasion, angiogenesis, and the expression of VEGF, IL-6, CSC genes, and miR-21 and miR-210 in PCa cells under hypoxic condition. We also investigated the role of miR-21 in the regulation of the expression of VEGF, IL-6, CSC marker genes, and their association with the formation of prostaspheres in PCa cells under hypoxic conditions. Furthermore, we examined the effects of a novel curcumin-derived synthetic analogue (CDF) that showed anti-tumor activity with a greater systemic and target tissue bioavailability, on cell survival, migration, invasion, angiogenesis, formation of prostaspheres, and the expression of HIF-1α, VEGF, IL-6, CSC marker genes, and miRNAs in PCa cells under hypoxic conditions. We found that hypoxia led to increased expression of VEGF, IL-6, and CSC marker genes such as Nanog, Oct4 and EZH2, and also increased the expression of miR-21 in human PCa cells. The treatment of cells with CDF inhibited the productions of VEGF and IL-6, and down-regulated the expression of Nanog, Oct4 and EZH2 mRNAs, as well as miR-21 in these cells under hypoxic condition. CDF also decreased cell migration of PCa cells under hypoxic condition. From these results, we conclude that the anti-tumor effect of CDF is in part mediated through deregulation of tumor hypoxic signaling pathways.

## Materials and Methods

### Cell Culture, Drugs and Reagents

Human prostate cancer cell lines PC-3 and LNCaP cells were maintained under standard culture condition (21% O_2_ and 5% CO_2_, 37°C). Hypoxic (1% O_2_) and 5% CO_2_ conditions were generated by controlling the input flow rates of nitrogen and carbon dioxide, respectively in the culture incubator. All the cell lines were maintained in 10% FBS-RPMI-1640 medium under standard cell culture condition. CDF was synthesized as described in our earlier publications [20]; [21].

### Cell Survival Assay

In order to investigate the effect of CDF on cell survival in human PCa cells under hypoxic condition, MTT assay was conducted using human PCa (PC-3 and LNCaP) cells. 3000 cells were plated in each well of the 96-well plates and incubated at standard culture conditions (21% O_2_ and 5% CO_2_) overnight. The cells were then treated with different concentrations of CDF (0.5 µM) and incubated for 8 h under hypoxic condition followed by 16 h under normoxic condition each day. After 3 days of treatment, the cells were harvested for the standard MTT assay, as described in our previous publications [22]; [23]. Each experiment was conducted in four replicates and repeated twice independently.

### Clonogenic Assay

Clonogenic assay was conducted to examine the effect of CDF on cell growth of PCa cells under hypoxic condition, as described previously [22]. Briefly, 5×10^4^ cells were plated in a six-well plate and after 3 days of exposure to 0.5 µM of CDF (8 h of hypoxic condition and 16 h of normoxic condition each day), the cells were trypsinized, and 1,000 single viable cells were plated in 100-mm Petri dishes. The cells were then incubated for 10 to 12 days at 37°C in a 5% CO_2_/5% O_2_/90% N_2_ incubator. Colonies were stained with 2% crystal violet, washed with water, and counted. Each experiment was conducted in three replicates and repeated twice independently.

### Invasion Assay

The *in vitro* invasion assay of PCa cells was conducted under hypoxic conditions by using Costar Transwell 24-well-plates with polycarbonate membrane (Corning Incorporated, Corning, NY), as described previously [23]. Briefly, 4×10^4^ of cancer cells (PC-3 and LNCaP) exposed to 3 days of incubation under normoxic or hypoxic condition were seeded into each well of the Matrigel pre-coated Transwell plates. The bottom wells of the system were filled with complete medium. After 20 h of incubation either in the absence or presence of CDF (0.5 µM), the invaded cancer cells were stained with 4 µg/mL of calcein-AM (Invitrogen) in PBS solution at 37°C for 1 h, following the manufacturer’s manual. The photographs were taken using a fluorescent microscope. Each experiment was conducted in three replicates and repeated twice independently.

### Wound Healing Assay

In order to examine the effect of CDF on cell migration of PCa cells under hypoxic condition, we conducted wound healing assay, as described previously [24]. Briefly, when the PC-3 cells became 90–95% confluent, the wound was generated by scratching the surface of the plates with a pipette tip. The cells were then incubated in the absence and presence of CDF (0.5 µM) and were cultured under hypoxic condition for 4 h, followed by 16 h of normoxic conditions, and then photographed with a Nikon Eclipse TS100 microscope, as described previously [23]. Each experiment was conducted in three replicates and repeated twice independently.

### Tube Forming Assay

In order to examine the effect of CDF on angiogenesis *in vitro* in vascular endothelial cells under hypoxic condition, we conducted tube formation assay, as described previously [25]; [26]. Briefly, 3×10^4^ rabbit vascular endothelial cells were plated in each well of the Matrigel-pre-coated 96-well plate in 100 µL of 10% FBS-DMEM medium, and exposed to normoxic or hypoxic conditions for 4 h of incubation at 37°C, followed by 16 h of normoxic conditions. The photograph was taken at 4 h and 20 h, respectively. Each experiment was repeated twice independently.

### Expression of VEGF and IL-6

ELISA was conducted to examine the effect of CDF on hypoxia-induced expression of VEGF and IL-6 in PCa cells. The culture media from PCa cells under hypoxic or normoxic conditions for 16 h were harvested for the measurement of VEGF and IL-6 by using ELISA assay kits (R&D Systems), following the manufacturer’s manual. Each experiment was conducted in three replicates and repeated twice independently.

### Sphere Formation Assay

The sphere formation assay was conducted to examine the effect of CDF on the CSC self-renewal capacity of PCa cells under hypoxic conditions, as described previously [23]. Briefly, single cell suspensions of PCa cells were plated on ultra low adherent wells of a 6-well plate (Corning, Lowell, MA) at 1,000 cells/well in sphere formation medium (1∶1 DMEM/F12 medium supplemented with B-27 and N-2 (Invitrogen), and exposed to hypoxic condition every other day. After 7 days, the spheres termed as “prostaspheres" were collected by centrifugation (300×g for 5 min), and counted. The proportion of sphere-generating cells was calculated by dividing the number of prostaspheres by the number of seeded cells with the diameter greater than 50 µmeters. Each experiment was conducted in three replicates and repeated twice independently.

### Immunostaining Assay and Confocal Microscopy

Single cell suspensions of PCa cells were plated on ultra low adherent wells of 6-well plate (Corning, Lowell, MA) at 10,000 cells/well in sphere-formation medium, and incubated for 24 h followed by culturing under hypoxic conditions every other day, as described above. After 7 days of drug treatment, 3 wells of the prostaspheres in each treatment group were pooled and collected by centrifugation (300×g for 5 min), washed with 1×PBS, and fixed with 3.7% parformaldehyde for 10 min at room temperature. Monoclonal CD44 and EpCAM antibodies (Cell Signaling) were used for immunostaining assay, following the manufacturer’s protocol, as described previously [24]; [27]. The CD44 or EpCAM labeled prostaspheres were photographed under a Nikon ESLIPSE E800 with 100x magnification. Confocal microscopy (Leica TCS SP5) was conducted in MIRL Core facility, Wayne State University School of Medicine. Each experiment was repeated twice independently.

### Transient and Stable Transfection of PCa Cells with cDNAs and miRNAs

The transfections of cDNAs and miRNAs were conducted by using ExGen 500 transfection reagent (Fermentas, Germany) and DharmaFECT transfection reagent (Dharmacon), respectively, following manufacturers’ manuals, as described previously [24]. The stable transfection of cDNAs were conducted under the selection of G418-containing medium (Sigma) and verified by Western blot analysis, as described previously [25]. Each experiment was repeated twice independently.

### Protein Extraction and Western Blot Analysis

Western blot analysis was conducted to measure the relative levels of HIF-1α protein in PCa cells under hypoxic conditions. Total cell lysates of the cells exposed to 16 h of hypoxic condition were obtained by lysing the cells in protein lysis buffer containing 50 mM Tris-HCl, 150 mM NaCl, 1% NP-40, 0.1% SDS, 0.5% sodium deoxycholate, 2 mM sodium fluoride, 2 mM Na_3_VO4_2_, 1 mM EDTA, 1 mM EGTA, and 1× protease inhibitor cocktail (Roche Diagnostics, Germany), and Western blotting was performed as described previously [24], and the signal intensity was measured using chemiluminescent detection system (Pierce Rockford, IL). Each experiment was repeated twice independently.

### Real-time (RT) Reverse Transcriptase-polymerase Chain Reaction (PCR) for Measuring the Expression of mRNAs and miRNAs

To determine the mRNA expression, two micrograms of total RNAs extracted from each sample were used for RT reaction in 20 µL of reaction volume using a reverse transcription system (Invitrogen) according to the manufacturer’s instruction. SYBR Green Assay kit (Applied Biosystems, Carlsbad, CA) was used for real time PCR reaction, using AB StepOnePlus Real-Time PCR System (Applied Biosystems), following manufacturer’s protocol. Sequences of PCR primers were described previously [23]. Data were analyzed using C_t_ method and were normalized by GAPDH expression in each sample. To determine the expression of miRNAs in the cells, the TaqMan MicroRNA Assay kit (Applied Biosystems) was used following manufacturer’s protocol. Total RNA was extracted from the cells and 5 ng of RNA was reverse transcribed as described earlier [28]. The miRNA primers were obtained from AB Systems. Real-time PCR reactions were then carried out in a total volume of 10 µL reaction mixture as described earlier [24], using StepOnePlus Real Time PCR System (AB Systems). Data were analyzed using C_t_ method and were normalized by RNU48 expression in each sample. Each experiment was conducted in three replicates and repeated twice independently.

### Luciferase Reporter Gene Assay

In order to examine the effect of CDF or miR-21 deficiency on miR-21 binding activity to 3′-UTR in PCa cells under hypoxic condition, we conducted miR-21-mediated luciferase reporter gene assay in PC-3 cells by using miR-21-mediated luciferase reporter gene vector (Signosis, Sunnyvale, CA), in which miR-21 binding to its DNA binding site at the luciferase gene vector suppresses the luciferase activity. Briefly, 10^4^ PC-3 cells were seeded in each well of the 96-well plates, and incubated overnight at the standard culture condition. The cells were then transfected with miR-21-suppressed luciferase reporter gene vector (Signosis, Sunnyvale, CA) by using ExGen 500 transfection reagent (Fermentas, Germany) or co-transfected with the luciferase vector and anti-miR-21 by using DharmaFECT transfection reagent (Dharmacon) following the manufacturer’s protocol, as described above. After overnight of transfection, the transfectants were treated with CDF for another 20 h under standard culture condition, and exposed to 4 h of hypoxic condition. Finally, the transfectants were harvested for luciferase activity assay by using Luciferase Assay System (Promega), following the manufacturer’s manual. Each experiment was repeated twice independently.

### Statistical Methods

Comparisons of treatment outcome were tested for significant difference by the paired *t* test. Statistical significance was assumed at a *P* value of less than 0.05.

## Results

### Effect of CDF on Cell Survival and Clonogenicity of PCa Cells Under Hypoxic Condition

**Figure 1:**
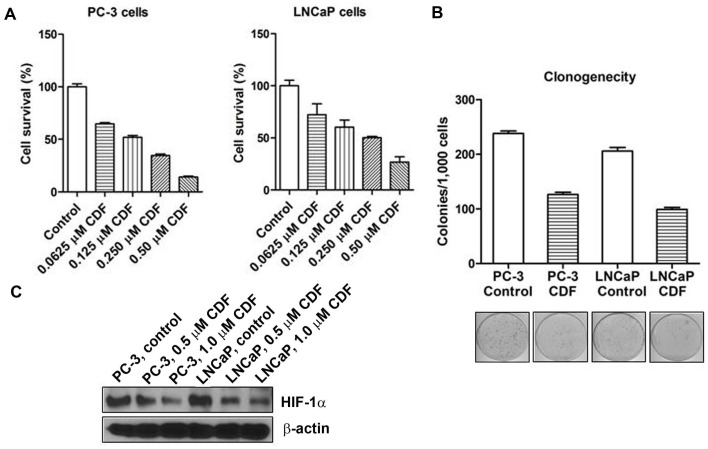
Effect of CDF on cell survival, clonogenicity, and the expression of HIF-1α protein in human prostate cancer (PCa) cells under hypoxic condition. The panels A, B, and C represent the data of cell survival, clonogenicity, and Western blot analysis, respectively. The bars in the figures indicate standard deviation of n = 4.

The data from MTT assay indicate that CDF remarkably inhibited cell survival of PC-3 and LNCaP cells under hypoxic conditions in a dose-dependent manner (Figure 1A). CDF treatment also decreased clonogenicity of PC-3 and LNCaP cells under hypoxic condition (Figure 1B). These finding suggested that CDF could inhibit cell survival and clonogenic growth of PCa cells under hypoxic conditions.

### Effect of CDF on HIF-1α Protein and the Productions of VEGF and IL-6 in PCa Cells Under Hypoxic Condition

**Figure 2:**
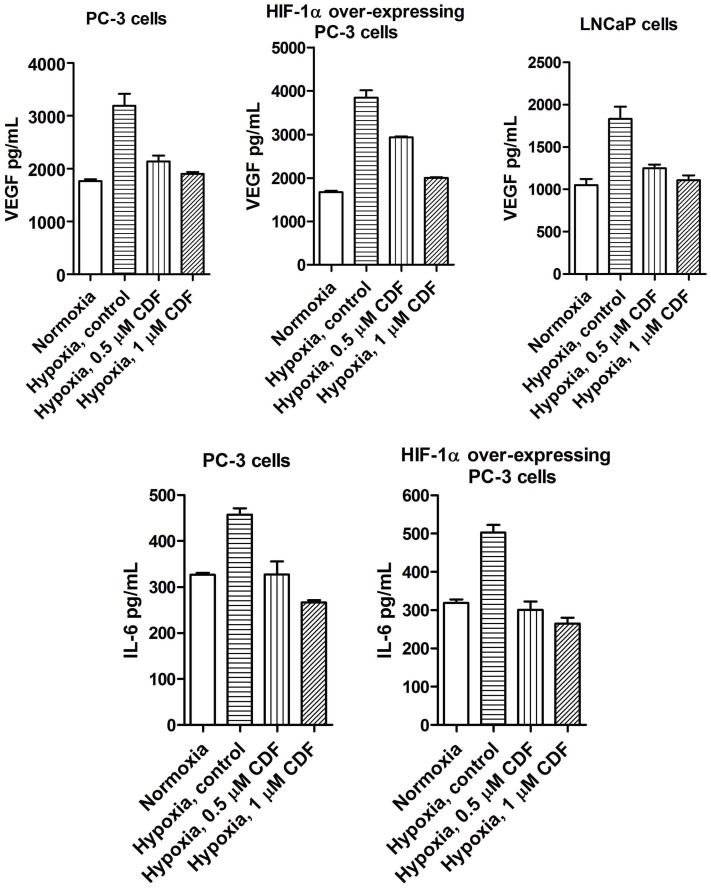
Effect of CDF on the productions of VEGF and IL-6 in human PCa cells under hypoxic condition. The conditioned media were collected from cells cultured under normoxic and hypoxic conditions as described under the methods section. The measurements of VEGF and IL-6 were conducted by ELISA. The bars in the figures indicate standard deviation of n = 3.

As shown in Figure 1C, CDF treatment decreased the relative level of HIF-1α in PC-3 and LNCaP cells under hypoxic condition. The cells incubated under hypoxic condition led to increased VEGF production, compared to the cells incubated under normoxic condition (Figure 2). CDF treatment remarkably decreased the production of hypoxia-induced VEGF in PCa cells (Figure 2). HIF-1α over-expressing PC-3 cells showed increased VEGF production under hypoxic condition, compared to its parental PC-3 cells. CDF treatment also inhibited the hypoxia-induced VEGF production in HIF-1α over-expressing PC-3 cells. These results suggest that the cells cultured under hypoxic condition leads to increased IL-6 production, compared to the cells incubated under normoxic condition (Figure 2). CDF treatment remarkably decreased the production of hypoxia-induced IL-6 in PCa cells (Figure 2). CDF also decreased hypoxia-induced IL-6 production in HIF-1α over-expressing PC-3 cells (Figure 2).

### Effect of CDF on Angiogenesis *in vitro* in Vascular Endothelial Cells Under Hypoxic Condition

**Figure 3:**
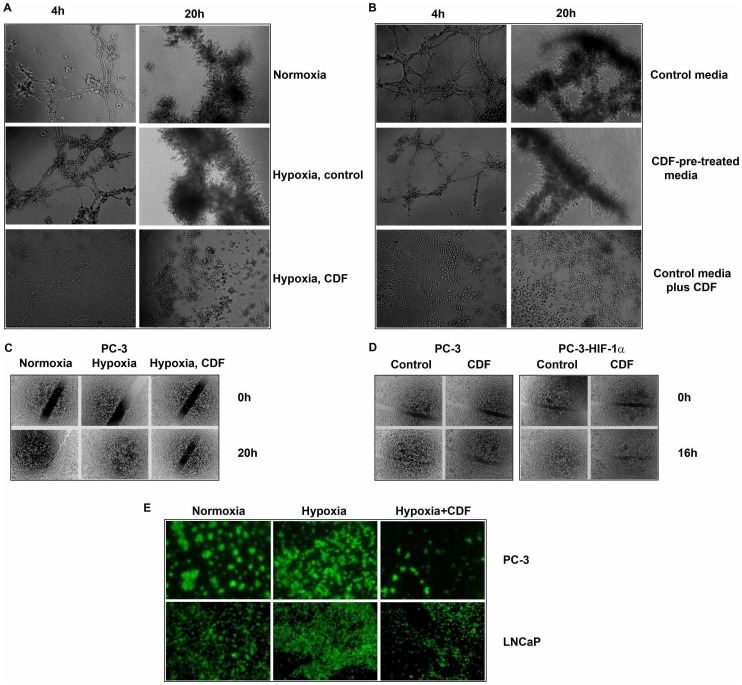
Effect of CDF on angiogenesis, cell migration, and invasion of PCa cells under hypoxic condition. The panels A&B, C&D, and E represent the data of angiogenesis *in vitro*, cell migration, and invasion. As described under the Methods section, angiogenesis *in vitro* was evaluated by the tube formation assay; cell migration was evaluated by wound healing assay; invasion was evaluated by chamber invasion assay.

Our results indicate that hypoxic conditions increased the tube formation capacity of vascular endothelial cells at 4 h and 20 h of incubations, respectively, compared to normoxic condition. CDF treatment inhibited the hypoxia-induced tube formation in vascular endothelial cells (Figure 3A). To clarify whether or not CDF-mediated molecules or CDF itself contributes to the inhibition of tube formation, we collected non-CDF-treated (control) and CDF-treated condition media from cancer cells and conducted the tube formation assay under normoxic condition. We found that the vascular endothelial cells incubated with control condition media had increased tube formation at 4 h and 20 h, compared to the cells incubated with CDF-pre-treated condition media. Addition of CDF to the control condition media significantly inhibited the tube formation, compared to the cells incubated at control condition media and CDF-pre-treated condition media (Figure 3B). These data suggest that CDF itself contributes to the inhibition of tube formation.

### Effect of CDF on Cell Migration and Invasion *in vitro* in PCa Cells Under Hypoxic Condition

Hypoxia-exposed PC-3 cells had increased capacity of wound healing, compared to the cells cultured under normoxia (Figure 3C). CDF treatment inhibited the wound healing capacity of PCa cells under hypoxic condition (Figure 3C and D). As shown in Figure 3D, over-expression of HIF-1α increased the wound healing capacity of PC-3 cells exposed to 16 h of hypoxic condition. CDF treatment inhibited the wound healing capacity in both HIF-1α-over-expressing PC-3 cells under hypoxic condition (Figure 3D). These results provided convincing data showing that CDF could inhibit hypoxia-induced cell migration of PCa cells, even in HIF-1α-over-expressing PCa cells. The *in vitro* invasion assay shows that both PC-3 and LNCaP cells exposed to hypoxic condition had increased capacity of invasion, compared to those cells exposed to normoxic condition (Figure 3E). CDF treatment inhibited the capacity of hypoxia-induced invasion of PCa cells.

### Effect of CDF on Gene Expression of CSC Markers and miRNA Expression in PCa Cells Under Hypoxic Condition

**Figure 4:**
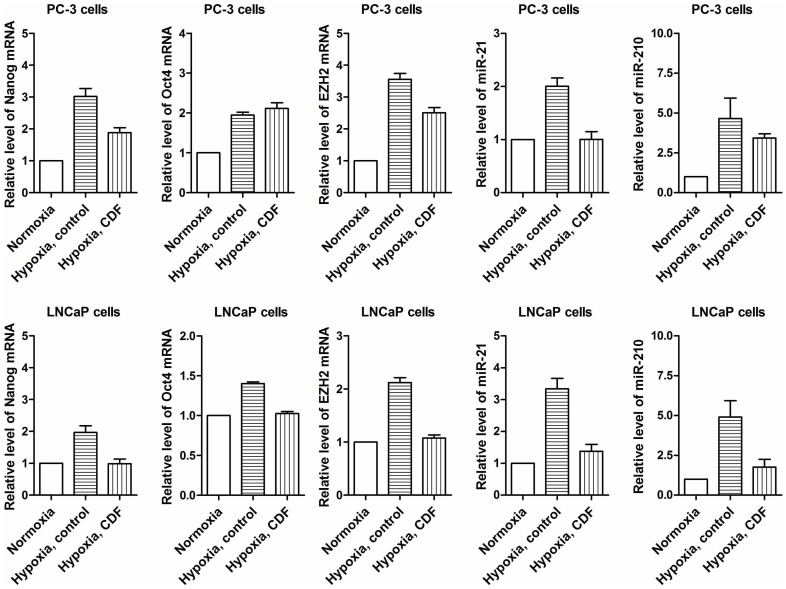
Effect of CDF on the expression of Nanog, Oct4, EZH2, miR-21 and miR-210 in PCa cells cultured under hypoxic conditions. Real time RT-PCR was conducted as described under the Methods section. The bars in the figures indicate standard deviation of n = 3.

The data from real-time RT-PCR assay indicate that hypoxia induced the relative levels of Nanog, Oct4 and EZH2 mRNAs as well as miR-21 and miR-210 in PC-3 and LNCaP cells whereas CDF decreased the levels of Nanog, Oct4 and EZH2 mRNAs as well as miR-21 and miR-210 in PCa cells under hypoxic condition (Figure 4).

### Effect of CDF or Anti-miR-21 on CSC Self-renewal Capacity and Cell Surface Markers CD44 and EpCAM in Human PCa Cells Under Hypoxic Condition

**Figure 5:**
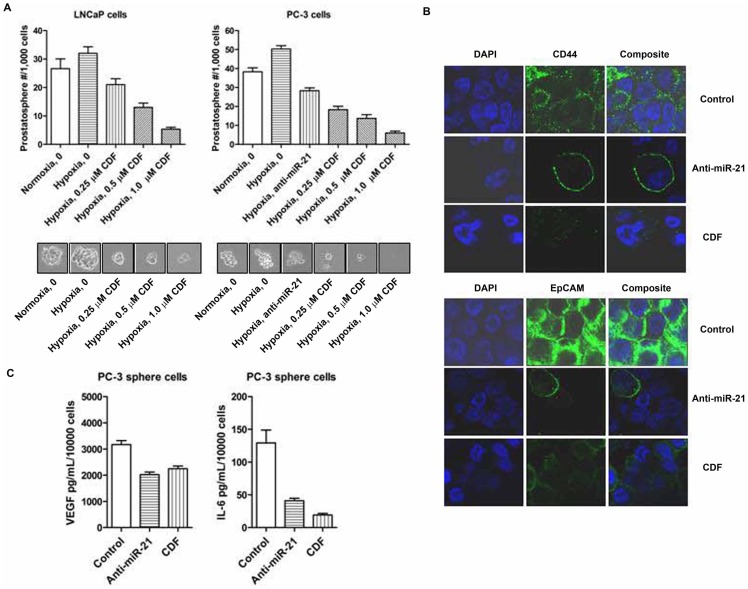
Effect of CDF and anti-miR-21 on the formation of prostaspheres, the expression of CD44 and EpCAM, and the production of VEGF and IL-6 in the CSC-like sphere-forming cells of PCa cells under hypoxic conditions. The panels A and B represent the data of the formation of prostaspheres, the expression of CD44 and EpCAM, and the production of VEGF and IL-6 in the CSC-like sphere-forming cells of PCa cells, respectively. The sphere formation assay was conducted to examine the self-renewal capacity of CSCs of PCa cells as described under the Methods section. Confocal microscopy was conducted to measure the expression of CSC surface markers CD44 and EpCAM in the sphere-forming cells derived from PCa cells as described under the Methods section. The bars in the figures indicate standard deviation of n = 3.

The data from the sphere formation assay indicate that anti-miR-21 decreased the formation of prostaspheres of PC-3 cells (Figure 5A). These data suggest that miR-21 may play an important role in the regulation of the self-renewal capacity of CSC-like PCa cells. CDF treatment also decesased the formation of prostaspheres of PCa cells under hypoxic conditions (Figure 5A). Moreover, we conducted confocal imaging microscopy for assessing the expression of CD44 and EpCAM in the sphere-forming cells of PC-3 cells. The results show that anti-miR-21 decreased the expression of CD44 and EpCAM in PC-3 sphere-forming cells under hypoxic condition, consistent with the CDF treatment (Figure 5B). These data suggest that CDF decreased the formation of prostaspheres and the expression of CD44 and EpCAM in part mediated by targeting the expression of miR-21.

### Effect of CDF or Anti-miR-21 on VEGF and IL-6 Production in the Sphere-forming Cells of PC-3 Cells Under Hypoxic Condition

We examined the effect of CDF on VEGF and IL-6 productions in the sphere-forming cells of PC-3 cells under hypoxic condition by ELISA assay. We found that PC-3 sphere-forming cells produced a larger amount of VEGF under hypoxic condition, compared to its parental PC-3 cells (3,172 pg/mL/10^4^ PC-3 sphere-forming cells vs 3192 pg/mL/10^6^ PC-3 cells; Figure 2 and 5C), suggesting that the PC-3 sphere-forming cells may promote angiogenesis by up-regulating the expression of VEGF production. We also found that CDF treatment decreased hypoxia-induced VEGF production in PC-3 sphere-forming cells, consistent with the results from the PC-3 sphere-forming cells with conditional inhibition of miR-21. Furthermore, we found that similar to VEGF production, the sphere-forming cells produced a remarkably higher amount of hypoxia-induced IL-6, compared to its parental cells (129.3 pg/mL/10^4^ PC-3 sphere-forming cells vs 457.6 pg/mL/10^6^ PC-3 cells; Figure 2 and 5C). CDF treatment or conditional deficiency of miR-21 by its inhibitor/siRNA decreased the production of hypoxia-induced IL-6 by the sphere-forming cells (Figure 5C). We also examined whether or not CDF treatment or miR-21 deficiency could regulate the gene expression of HIF-1α, VEGF, IL-6, CD44, EpCAM, and EMT markers in PC-3 sphere-forming cells under hypoxic condition. We found that conditional suppression of miR-21 resulted in a significant decrease in the relative mRNA levels of HIF-1α, VEGF, IL-6, CD44, EpCAM, and mesenchymal markers of the EMT phenotype such as ZEB1, Vimentin, and Twist, and increased the relative mRNA levels of epithelial marker E-cadherin in PC-3 sphere-forming cells under hypoxic conditions (data not shown). Similarly, CDF decreased the mRNA levels of HIF-1α, VEGF, IL-6, CD44, EpCAM, and mesenchymal markers ZEB1, ZEB2, Vimentin, and Twist in PC-3 sphere-forming cells under hypoxic condition. However, CDF also decreased the gene expression of E-cadherin in PC-3 sphere-forming cells under hypoxic condition (data not shown).

### Effect of CDF on the Expression of miRNAs in PC-3 Sphere-forming Cells Under Hypoxic Condition

**Figure 6:**
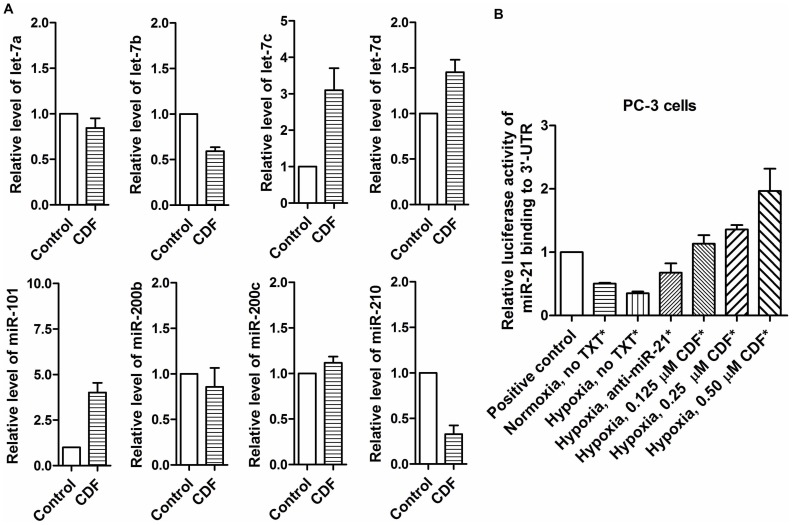
Effect of CDF and/or anti-miR-21 on miRNA expression and miR-21-mediated luciferase activity in human PCa cells under hypoxic conditions. The panels A and B represent the data of miRNAs in the CSC-like sphere-forming cells derived from human PCa cells and luciferase activities in PCa cells, respectively. The transfection of miR-21-mediated reporter gene luciferase vector and anti-miR-21 were conducted in PC-3 cells, as described in detail under the Methods section. The luciferase activity was measured by using Promega luciferase assay system kit, following the manufacturer’s manual. The bars in the figures indicate standard deviation of n = 3.

We examined the effect of CDF on Let-7, miR-21, miR-101, and miR-210 in PC-3 sphere-forming cells under hypoxic condition. The results showed that CDF increased the relative miRNA levels of let-7c,d, and miR-101 and decreased the relative level of miR-210 in PC-3 sphere-forming cells under hypoxic condition (Figure 6A). These data suggest that CDF could deregulate the expression of hypoxia-associated miRNAs in CSC-like cells of PCa cells.

### Effect of CDF Treatment or miR-21 Deficiency on miR-21 Binding Activity to 3′-UTR in PCa Cells Under Hypoxic Condition as Assessed by Luciferase Assay

We examined the effect of CDF treatment or miR-21 deficiency on miR-21 binding activity to 3′-UTR in PCa cells under hypoxic condition using miR-21-mediated luciferase reporter gene assay. As shown in Figure 6B, we found that hypoxia decreased the luciferase activity in the PC-3 cells transfected with miR-21-mediated luciferase vector, compared to the same cells under normoxic condition, suggesting that hypoxia increased the miR-21 binding activity, leading to the decrease in the luciferase activity. Anti-miR-21 increased the luciferase activity in PC-3 cells under hypoxic condition, compared to the same cells without treatment, suggesting that anti-miR-21 decreased the miR-21 DNA binding, leading to an increase in the luciferase activity in PC-3 cells under hypoxic condition. Similarly, CDF treatment showed increased luciferase activity in PC-3 cells under hypoxic condition in a dose-dependent manner, suggesting that CDF could inhibit the miR-21 DNA binding in PC-3 cells.

## Discussion

A number of epidemiological and clinical studies have shown that hypoxia and hypoxia-induced signaling pathways are associated with poor prognosis of patients diagnosed with solid tumors including prostate cancer (PCa). Emerging evidence also suggest that hypoxia increases cell migration, invasion, and angiogenesis, leading to tumor aggressive phenotypes. Here, we confirm that hypoxia induces cell migration, invasion, and angiogenesis, and increased the production of VEGF in PCa cells. We also observed that hypoxia induces the formation of prostaspheres in PCa cells, consistent with increased expression of CSC markers genes such as Nanog, Oct4, EZH2, CD44, and EpCAM in PCa cells. It has been shown that hypoxia plays a key role in the regulation of CSC characteristics through HIF proteins, and HIF downstream target genes such Oct4 and Notch-1 [6]; [7]. These findings suggest that hypoxia may play a key role in the regulation of CSC characteristics, leading to tumor aggressive phenotype.

Emerging evidence suggests that miRNAs play critical roles in the development and progression of tumors through regulation of mRNA degradation or protein translation mediated through their binding to the 3′ untranslated region (3′-UTR) of the target genes. It has been documented that miR-21 is considered a pro-oncogenic molecule, which promote tumorigenesis. The clinical studies has shown that increased expression of miR-21 is associated with poor clinical prognosis in a wide variety of cancers including PCa [29]; [30]. It has been reported that increased expression of miR-21 results in the decreased expression of PTEN, a known tumor suppressor in cancers [22]; [24]. The miR-21 has also been reported to show anti-apoptotic, proliferative, invasive and angiogenic properties in cancer cells [12]; [14]; [30]. Increased expression of miR-21 has also been reported in cancer cells such as breast cancer cells under hypoxic conditions [16]; [31]. Over-expression of miR-21 in DU145 cells increased the expression of HIF-1α and VEGF, leading to increased tumor angiogenesis [32]. Over-expression of miR-21 target gene, PTEN, inhibits tumor angiogenesis through the down-regulation of HIF-1α and VEGF in cancer cells [32]. Thus, HIF-1α is a key downstream target of miR-21 in the regulation of tumor angiogenesis. It has also been shown that over-expression of miR-21could promote the survival of mesenchymal stem cells in bone marrow under hypoxic condition, and down-regulation of miR-21 increases apoptosis of mesenchymal stem cells [33]. Here we report, for the first time, showing that hypoxia leads to increased expression of miR-21 in PCa cells, consistent with increased expression of VEGF, and increased self-renewal capacity of CSC-like cells. The knock-down of miR-21 expression by its siRNA inhibitor decreased the expression of VEGF, CD44, EpCAM, and the CSC self-renewal capacity of PCa sphere-forming cells under hypoxic condition, which suggest that hypoxia-induced miR-21 expression plays a critical role within the tumor microenvironment, contributing to tumor aggressiveness.

The miR-210 has been identified as hypoxia-induced miRNAs in a variety of cells including cancer cells through large number of experimental studies [16]; [19]; [34]–[41]. Clinical studies have also shown that the increased expression of miR-210 is significantly associated with poor prognosis in PCa and other cancers [19]; [38]; [42]. Several experimental studies have shown that the hypoxia-induced miR-210 increases the expression of hypoxia-induced downstream targets VEGF and CAIX (carbonic anhydrase 9) in pancreatic cancer cells by a HIF-1α-dependent mechanism [19]; [36], suggesting a regulatory role in tumor angiogenesis [16]–[18]; [43]–[45]. Hypoxia-induced miR-210 has also been shown to modulate DNA repair pathway. Forced over-expression of miR-210 was found to suppress the levels of radiation sensitive 52 (RAD52), a key factor in homology-dependent repair (HDR), leading to impaired DNA repair and genetic instability [46]. In our current study, we have demonstrated that hypoxia increased the expression of miR-210 in PCa cells and CSC-like sphere-forming cells. These data suggest that hypoxia-induced miR-210 plays an important role in the tumor microenvironment, suggesting that targeting miR-210 would provide a novel therapeutic strategy for the treatment of human malignancies including PCa.

Inflammatory cytokine IL-6 has been known to play a critical role in the development and progression of tumors including PCa [47]–[49]. The clinical data showed a strong association between increased expression of IL-6 and poor outcome of patients diagnosed with a variety of tumors including PCa [47]–[49]. The experimental studies have demonstrated that IL-6 could promote tumorigenesis, angiogenesis, and metastasis [50]; [51]. IL-6 is also known to induce multi-drug resistance to chemotherapy [52]; [53]. Moreover, emerging evidence suggest that IL-6 may have an important role in the CSC phenotype and function. One experimental study has shown that human lung CSC-derived tumor contained 2–3 higher levels of inflammatory cytokines including IL-6 [54], which is consistent with recent findings showing that the lung CSC-like cells have high level expression of IL-6/IL-6R [52]. It has also been shown that IL-6 treatment promotes the formation of mammosphreres of human breast cancer and normal mammary gland epithelial cells, consistent with the activation of Notch signaling [47]. Additionally, IL-6 has been found to enhance the tumorigenicity in glioblastoma, consistent with increased capacity of CSC self-renewal [55]; [56]. Consistent with these findings, our results showed for the first time that the CSC-like sphere-forming cells of PCa could produce a significantly higher amount of IL-6, compared to its parental non-sphereforming cells under hypoxic condition. These findings suggest that IL-6 may act as a direct regulator of the self-renewal capacity of CSCs; however, the exact role of IL-6 in the regulation of CSC characteristics is not fully understood.

In our previous studies, we have shown the superiority of a novel synthetic analogue of curcumin referred to as Difluorinated-Curcumin (CDF) [20]; [21]. Moreover, we have also reported that CDF could function as a potential anti-tumor agent against human pancreatic tumor *in vitro* and *in vivo* mediated via regulation of miRNAs, CSC phenotype and function, and deregulation of multiple cellular signaling pathways such as NF-κB, Akt, COX-2, Notch-1, and EZH2 [22]–[24]; [57]. In the current study, we showed that CDF can inhibit cell survival, clonogenicity, migration, invasion and angiogenesis, and the CSC self-renewal capacity of human PCa cells under hypoxic conditions, which is consistent with inhibition in the expression of miR-21, miR-210 and HIF-1α, and the CSC marker genes. These findings provided strong evidence in support of the potential role of CDF as an anti-tumor agent mediated by targeting multiple signaling pathways including hypoxia-induced CSC phenotype and function, which could be relevant within the tumor microenvironment *in vivo*, and consequent down-regulation of tumor aggressive phenotype of PCa.
